# Engaging Religious Institutions and Faith-Based Communities in Public Health Initiatives: A Case Study of the Romanian Orthodox Church During the COVID-19 Pandemic

**DOI:** 10.3389/fpubh.2021.768091

**Published:** 2021-12-16

**Authors:** Stefan Dascalu, Patrik G. Flammer, Mahan Ghafari, Shaun C. Henson, Roger Nascimento, Michael B. Bonsall

**Affiliations:** ^1^Department of Zoology, University of Oxford, Oxford, United Kingdom; ^2^Avian Influenza Research Group, The Pirbright Institute, Woking, United Kingdom; ^3^Ian Ramsey Centre for Science and Religion, University of Oxford, Oxford, United Kingdom; ^4^Faculty of Theology and Religion, University of Oxford, Oxford, United Kingdom; ^5^Centre for Tropical Medicine and Global Health, University of Oxford, Oxford, United Kingdom

**Keywords:** COVID-19, public health, religion, Romanian Orthodox Church, Romania, faith-based communities, religious institutions, religious leaders

## Abstract

The success of public health interventions is highly dependent on the compliance of the general population. State authorities often implement policies without consulting representatives of faith-based communities, thereby overlooking potential implications of public health measures for these parts of society. Although ubiquitous, these challenges are more readily observable in highly religious states. Romania serves as an illustrative example for this, as recent data identify it as the most religious country in Europe. In this paper, we discuss the contributions of the Romanian Orthodox Church (ROC), the major religious institution in the country, to the national COVID-19 mitigation efforts. We present not only the positive outcomes of productive consultations between public health authorities and religious institutions but also the detrimental impact of unidirectional communication. Our work highlights that an efficient dialogue with faith-based communities can greatly enhance the results of public health interventions. As the outlined principles apply to a variety of contexts, the lessons learned from this case study can be generalized into a set of policy recommendations for the betterment of future public health initiatives worldwide.

## Introduction

The COVID-19 pandemic created an unprecedented public health emergency. Across the world, the responses of governments varied in terms of effectiveness and timeliness. In many countries, healthcare systems became overwhelmed by the high number of infections and hospitalizations. Appropriate public health interventions such as social distancing, lockdowns, and self-isolation have proven effective in controlling the spread of the disease and the resurgence in cases before the implementation of mass-vaccination programmes (1, 2). During these times, efficient communication from public health authorities became an essential criterion for both tackling misinformation about the pandemic and reducing social unrest caused by restrictive measures (3, 4). Moreover, when COVID-19 vaccination campaigns unfolded, accessing all available channels of communication became paramount for providing appropriate information to the public and thus achieving successful outcomes (5–7).

Public health interventions are reliant on various factors, including the nature of the implemented measures and the trust and compliance of the public. In the context of healthcare emergencies, the timeliness of the response is crucial, and this imposes additional challenges for achieving the desired outcomes (8–10). Importantly, emergency situations require efficient and extensive dialogue with the public. As such, reaching beyond the usual information channels during crisis situations can greatly facilitate the success of public health measures. These communication methods include mechanisms such as engaging non-governmental entities, influential public figures, and religious communities.

The involvement of religious institutions and faith-based organizations in public health campaigns is known to have a great potential for ensuring the success of such initiatives (11, 12). Furthermore, the influence of religious leaders within their communities is an essential factor for increasing public acceptance of such campaigns and delivering successful outcomes (13–16). Indeed, during the COVID-19 pandemic, recommendations for religious leaders and faith-based organizations were issued by the WHO and other healthcare agencies (17, 18). These guidelines mainly concerned the nature of religious gatherings, specific rituals, and important religious ceremonies in order to minimize the risk of disease transmission. However, few, if any, comprehensive guidelines were given to state authorities about how to engage religious institutions and organizations for the betterment of public health. Unfortunately, on many occasions, the absence of efficient dialogue resulted in mixed responses from religious communities, causing people to question or even directly oppose the implementation of official COVID-19 mitigation policies.

These issues were not only identified in highly religious countries, but they were also present in faith-based communities from more secular states. However, the negative effects were more pronounced in the former, where religious leaders and faith-based institutions have a much stronger influence on the wider population (19). In Europe, eastern countries stand out with an overall higher degree of religiosity, often coinciding with Orthodox Christianity as the predominant religious affiliation (20). With this in mind, recent data identify Romania as the most religious country in Europe, with more than half of its citizens being “highly religious” (20). Roughly 81.9% of Romanians are Orthodox Christians, 6.4% are Protestant Christians, 4.3% are Roman Catholic, and 1.1% are of other religions (Islam, Judaism, or other Christian denominations) (21).

As the established church of the country, the Romanian Orthodox Church (ROC) ranks second in terms of overall public trust, after the military (22, 23). As such, the responses of the ROC during the COVID-19 pandemic provide valuable insights into how a religious institution can contribute to public health efforts during a situation of crisis. In the following sections, we provide a descriptive analysis of the COVID-19 responses of the Romanian authorities and the ROC, emphasizing the importance of bidirectional communication during public health interventions. We argue that the absence of efficient dialogue can create misunderstandings which negatively influence the outcomes of the implemented measures. By contrast, if religious leaders and faith-based communities are involved in active consultations with state authorities, the effects of the implemented public health policies can be substantially enhanced.

## Church Contributions To The Management of COVID-19

### Public Trust and Compliance During the Early Stages of the Pandemic

During the early days of the pandemic, Romania faced an unprecedented influx of members of its diaspora, which ranks among the largest in the world (23). To prevent an uncontrolled outbreak across the country, state authorities quickly implemented self-isolation and quarantine measures for people coming from abroad. The public perception of this situation was not favorable, especially when quarantine centers were opened in localities close to border crossing points. Returning Romanians were often vilified on social media platforms for importing infections into the country, and there were even protests outside quarantine centers, demanding the closure of these facilities (23). However, efficient communication from state authorities and the ROC mitigated the impact of these protests by increasing the understanding of the situation and advocating tolerance. Furthermore, high-ranking clergy of the ROC issued statements supporting the official guidelines, and some monasteries even provided quarantine spaces for people who were returning from abroad (24). These actions taken by the ROC during the early stages of the COVID-19 pandemic considerably bolstered the compliance of the public (23).

### Orthodox Easter

In March 2020, the authorities implemented a national lockdown due to rapidly increasing numbers of COVID-19 cases in Romania (23). This raised concerns about how the upcoming celebrations of Orthodox Easter would proceed. On the one hand, public health experts warned about the risks of superspreading events, particularly during mass gatherings which are customary to the Easter service. On the other hand, clergy and church officials were concerned about the state interfering with religious freedom and the right to worship. This situation created a conflict which needed to be addressed promptly, as tensions were already high due to other restrictions such as mandatory face covering, social distancing, and the national lockdown. After extensive communication between state officials and representatives of the ROC, the Easter services were allowed to proceed without public attendance. The distribution of the Holy Light to citizens' homes was performed by volunteers from local congregations, under the supervision and guidance of police officers and gendarmes. These extraordinary measures, only possible with the support of the ROC, were paramount to both preventing potential superspreading events and avoiding public unrest.

The following year, Orthodox Easter coincided with a period of epidemic decline and an increase in vaccination coverage (25). This led to an exception to the national curfew being granted for the public to attend the Easter service without any restrictions. Although this decision was welcomed by Orthodox believers and the ROC, it was met with criticism by various officials and public health experts. However, the Patriarchy issued guidelines for the safe officiation of the Easter service, including social distancing and other sanitary precautions that would limit the potential spread of infection (26). As in the previous year, these measures were the direct result of state authorities engaging in dialogue with representatives of the ROC. Consequently, contrary to the concerns that were voiced about the risk of allowing Easter services to proceed with full attendance, the average number of daily COVID-19 cases maintained a decreasing trend in the following weeks. An outcome similar to that of the previous Easter celebrations was achieved, although in a very different epidemiological context. In both situations, an efficient dialogue between state authorities and the ROC enabled the COVID-19 mitigation measures to be adapted to the current state of the pandemic, thereby greatly enhancing the overall outcomes of these public health actions.

### Charitable Work

The contribution of the ROC to the management of the pandemic was not limited to endorsing and abiding by the official public health guidelines. Like other religious institutions worldwide, the ROC was involved in substantial charitable and philanthropic activities (27). In just 1 month after the first COVID-19 infections were detected in Romania, the ROC spent over one million USD in donations in order to help manage the impact of the pandemic (28). The ROC also donated medical devices and equipment to healthcare centers across the country. Moreover, in cooperation with regional and national authorities, faith-based organizations of the ROC created emergency hotlines to assist the medically and socially vulnerable. Together, the charitable activities of the Church have greatly contributed to mitigating the societal impacts of COVID-19 during all the stages of the pandemic.

### Vaccination

In general, the official stance of the ROC toward vaccination is supportive, subject to individual rights being respected and an absence of commercial motivation (29). However, on many occasions, various faith-based organizations and highly influential figures of the ROC have opposed vaccination and have contributed to the spread of misinformation. As these activities were never officially condemned by the ROC, many religious leaders adopted a rather impartial stance on vaccination, probably because of its polarizing nature within their communities. Thus, there was an urgent need for issues concerning vaccine acceptance and misinformation to be addressed, even before the national COVID-19 vaccine rollout. Indeed, state authorities were aware that support from the ROC would have greatly benefited the national immunization campaign (27).

On 14 December 2020, before the approval of any COVID-19 vaccines, formal discussions took place between state officials, public health experts, and representatives of the major religions in Romania (30). It was agreed that health authorities would provide religious institutions with comprehensive information about COVID-19 vaccination. Several days after these discussions, the spokesperson of the ROC issued a statement in which a distanced and undecided stance was communicated on behalf of the Romanian Orthodox Patriarchate (31). However, dialogue continued between public health specialists and various representatives of the ROC, including the clergy. These activities used the already-established channels of communication with the ROC described in the examples above. Importantly, concerns about vaccination that were grounded in religious arguments were identified and addressed promptly with appropriate scientific information. As a result, religious leaders of the ROC and influential figures of local faith-based organizations were acclimatized to important concepts regarding COVID-19 vaccines and were given argumentative resources to combat misinformation.

Nearly 1 month after the meeting with the state authorities and the public health experts, religious representatives received informative materials about COVID-19 vaccination in the form of a comprehensive brochure (32). Shortly thereafter, the information was circulated to all clergy of the ROC alongside a statement from Patriarch Daniel of Romania. In this announcement, the national vaccination campaign was endorsed and the importance of verified and accessible sources of information about COVID-19 vaccines was emphasized (32). Furthermore, for the first time, a broadcast dedicated entirely to immunization was aired on the television channel of the ROC, where religious concerns about vaccines were addressed with scientific arguments (33). Subsequently, religious leaders and clergy of the ROC from across the country issued statements in favor of the national immunization campaign (27). As such, people were urged to trust the public health authorities and to use verified sources of information to answer questions about vaccination. Respectful consultation and cooperation thus enabled the vaccination campaign to use the extensive networks of the ROC for communicating public health information.

These actions were likely beneficial for the COVID-19 vaccination campaign, as the messages were able to reach a significant segment of the population. However, by October 2021, Romania had one of the lowest COVID-19 vaccination coverages and some of the highest COVID-associated mortalities in Europe (34). Furthermore, many voices within the ROC still actively opposed vaccination and religious arguments continued to be misused for discouraging vaccination. This phenomenon, however, can be explained by the high level of autonomy of the parishes, bishoprics, and archbishoprics within the ROC. Nonetheless, the overall support from the ROC was inadequate, and the fact that influential bishops did not vaccinate themselves probably contributed to the persistence of mistrust toward COVID-19 vaccines (34). Although difficult to assess, engaging representatives of the ROC in active dialogues and consultations with state authorities from the earlier stages of the pandemic might have improved this situation. Indeed, the first public discussion on vaccination involving representatives of the major religions in Romania (including the ROC) took place as late as October 2021 and was organized by an online news agency (35). During the discussions, the lack of consultations regarding the implementation of COVID-19 public health policies was highlighted by all the religious representatives present. Such public dialogues would have likely improved the trust of faith-based communities in state authorities, thereby increasing COVID-19 vaccine acceptance of both clergy and believers.

## The Importance of Bidirectional Communication

### Religious Gatherings and the Spread of COVID-19

In light of the measures that were implemented in Europe to limit the number of COVID-19 cases, religious institutions were faced with the difficult decision of whether or not to abide by the restrictions that often directly interfered with very important rituals and traditions (19). Although major religions generally supported the country-specific epidemic control measures, there were situations when these were challenged or not followed altogether by various religious communities. One of the most dramatic examples concerns the death of Patriarch Irinej of Serbia (36). He became infected with COVID-19 after officiating the funeral of Metropolitan Amfilohije of Montenegro, who succumbed to the same illness after holding religious ceremonies where public health guidelines were disregarded. In Romania, a similar scenario occurred when Archbishop Pimen passed away after being diagnosed with COVID-19. Pimen, the oldest member of the ROC synod, likely acquired the disease after coming into contact with infected staff or clergy from his archbishopric (37). Another possibility was also put forward after a video of him officiating the Easter service without respecting social distancing measures was released by the press (38). Regardless, his funeral attracted thousands of Christians and, contrary to the plea from the ROC that the social distancing measures should be respected, many people disregarded the official guidelines (37, 39).

Another dramatic situation concerning large gatherings of believers not abiding by social distancing and other epidemic control measures was at the pilgrimage of Saint Parascheva in 2020. In previous years, over the course of a few days, this event attracted close to 100,000 Orthodox Christians to the city of Iaşi, where the relics of the Saint are housed (40). This caused substantial pressure on state officials from both believers and clergy to allow the religious event to proceed. Consequently, the decision was made that the ceremonies would be permitted, but with participation restricted to the residents of Iaşi (41). Although this measure was intended to discourage traveling in order to limit the potential for COVID-19 transmission, people from all over Romania came to Iaşi to attend the religious services. There, unable to enter the premises of the cathedral where the ceremonies were taking place, large gatherings formed to protest the decision of the authorities (42). As a consequence of public pressure, the believers were eventually allowed entry to the relics of St Parascheva. This not only caused crowding at the pilgrimage site and an increased risk of transmission but also severely undermined the authority of the state officials. Despite the efforts of the ROC to implement sanitary measures (e.g., providing disinfectants) and enforce social distancing at the event, the failure of the authorities to control the flow of people negated all positive intentions to reduce the risk of infectious spread.

Throughout the COVID-19 pandemic, there were several other examples of pilgrimages where the official guidelines were not respected. Just as for the celebration of St Parascheva, the dialogue aimed at resolving potential issues was on many occasions non-existent, and government officials did not provide the necessary support to the ROC (43). As a result, attempts of the local authorities to enforce the COVID-19 measures were received with public outcries and protests. Such reluctance to abide by the control measures was generally grounded in the interpretation that the state is interfering with religious freedom (44).

### State Policy Consequences on Religious Rituals

Public health policies in response to the COVID-19 pandemic often impacted the ways in which religious ceremonies were officiated. As such, practices that traditionally involved large gatherings of people, such as regular church services, baptisms, or weddings, were limited to a maximum number of attendants or were temporarily banned when the number of COVID-19 cases was high (45, 46). Similarly, funerals were limited to the immediate family of the deceased, and special precautions (e.g., sealed coffins) were taken to limit the potential spread of the virus. With this in mind, the ROC issued recommendations early on to the clergy about how to adapt religious practices to the new COVID-19 safety requirements (47). Furthermore, in addition to limiting the number of people attending church services, the ROC implemented further safety measures which were not yet mandated by the state (47, 48).

Although the COVID-19 mitigation efforts were generally endorsed by the ROC, there are several examples at different stages of the pandemic where successful outcomes were not achieved. For instance, ample discussions took place with regard to changing the religious practice of Communion so that single-use plasticware would replace the traditional chalice and spoon ordinarily used for this sacrament (49). Eastern Christian tradition, along with Christian tradition more widely, holds that the Church is most authentically itself when the faithful gather together in worship (50). Importantly, Communion is the highest expression and practice of the conjoint set of religious acts, where believers receive the body and blood of Christ, personified by bread and wine, respectively. The chalice and spoon, alongside other symbols, are all understood to be important aspects of communing with God prayerfully and with one another faithfully. Important theological meaning thus underpins the statements made, the bodily gestures and postures enacted, and the ritualistic objects used.

From a public health perspective, several implications arise concerning Communion and the potential transmission of infectious agents (51). Firstly, large gatherings of people during any religious service may facilitate disease transmission. Secondly, the act of Communion involves proximal contact, as believers receive the sacrament from the priest. Moreover, Orthodox Christian tradition involves the shared use of the chalice and spoon among participating worshipers, without any disinfection during the ritual itself (52). Although there are no documented cases for disease transmission or outbreaks arising from partaking in Communion, scientific evidence suggests that infectious spread is possible (51). It is therefore understandable that concerns were raised regarding Communion during the early stages of the pandemic, especially as the infectious dynamics of COVID-19 were still unknown (53). However, the profound theological meaning of the act of Communion needed to be acknowledged if any public health policies were to directly or indirectly affect this religious practice.

In May 2020, a set of recommendations from the Romanian National Institute of Public Health (INSP) included measures to be implemented in places of worship. Importantly, the document contained the statement that “offering/receiving Holy Communion is to be avoided if disposable cups and spoons cannot be provided” (49). Although the purpose of this recommendation had a good justification given the potential for infectious transmission, it caused wide outrage among believers and clergy of the ROC. As Communion is central to Christian practice, many interpreted the guidelines issued by the INSP as the state interfering with freedom of religion (43). Moreover, a press release from the ROC stated that any decisions regarding Communion are to made exclusively by the Church and that the state should not interfere with the ways in which ceremonies are officiated (54). This example illustrates the importance of finding a common ground of discourse to address the implementation of public health interventions without causing profound theological disruptions. Indeed, many of the issues that occurred could have been avoided if the state authorities had initiated consultations with theologians and religious representatives.

### Clergy Opposition to COVID-19 Guidelines

The failure of state authorities to provide a plan for how and when the restrictions would be relaxed sometimes contributed to the reluctance of faith-based communities to endorse and abide by the measures (55). This situation is substantially different to countries where a systematic approach to the ease of restrictions was provided to religious institutions, which was based on the national, regional, and local evolution of COVID-19 cases (19). For example, the UK government had issued detailed guidelines about how religious practices are to resume both during televised press conferences and on the government's dedicated website (56). At the same time, the implemented policies were continuously adapted to the evolution of the pandemic and were clearly communicated to the public. Together, these approaches greatly increased overall compliance, and the desired outcomes for public health were successfully achieved. As such, religious institutions were virtually back to normal routines by summer 2021, with pandemic mitigation measures mostly being implemented on a voluntary basis.

In Romania, although the ROC officially supported the COVID-19 mitigation policies, the absence of guidelines ensuring the safe continuation of religious practices created a wide range of issues. Indeed, an official statement from the ROC requested clarity about the conditions that would allow for a safe resumption of normal religious services (57). This absence of a systematic plan to relax restrictions had already caused outcry from various clerics throughout the country, including some prominent figures of the church. The most notable example is that of Archbishop Teodosie, who called upon believers to attend a second Easter service more than 1 month after the date of this central Orthodox holiday (58). As such, Christians were urged to attend the ceremony and not respect governmental COVID-19 guidelines. It is important to note that the ROC neither endorsed nor condemned this second Easter service officiated by Teodosie, citing the autonomy of archbishoprics which allows for such decisions to be made independently of the Patriarchy (59). Although state authorities condemned the actions of Teodosie, no official sanctions were implemented, neither for him nor for the people that disregarded public health measures.

Throughout the pandemic, Archbishop Teodosie became one of the major advocates against public health measures that directly or indirectly influenced religious practices in Romania. There were numerous instances when his defiance of national COVID-19 policies received ample media coverage (58). These included summoning Christians to pilgrimages when large gatherings were forbidden, disregarding social distancing measures during church services, and even propagating misinformation about the COVID-19 pandemic (58, 60, 61). The consequences of Teodosie's actions not only attracted criticism from public health experts, but they also eventually caused the ROC to publicly condemn his behavior. As such, the request of Teodosie to become Metropolitan was denied, partly on the grounds of his stances against the official position of the Church during various stages of the COVID-19 pandemic (62). Furthermore, the letter, which was leaked to the press, stated that his actions were “an act of rebellion” and also brought forward plagiarism charges which Teodosie faces at the university where he serves as head of faculty (62).

In general, the ROC tried to dissociate itself from the instances when clergy disregarded official COVID-19 guidelines, and publicly condemned such actions. However, the absence of any sanctions from state authorities against these members of the clergy most likely delayed any actions from the ROC. Indeed, public pressure alone was not sufficient to trigger disciplinary measures against high-ranking clergy of the ROC. By contrast, continuous dialogue between state officials and the ROC would have most likely allowed for these issues to be addressed much more efficiently.

## Discussion

Religion is an important part of life for the majority of the Romanian population. We therefore analyzed the responses of the ROC to the COVID-19 pandemic and provided illustrative examples of the role of religious institutions during a major public health crisis. From the onset of the pandemic, the ROC supported national policies and encouraged the community to follow official guidelines. The ROC further introduced specific measures for adapting religious services and took an active stance against misinformation about both the pandemic and the national vaccination campaign. Furthermore, to assist those most affected by the pandemic, the ROC substantially increased its charitable activities. These actions, which significantly enhanced the national COVID-19 mitigation efforts, were enabled by efficient bidirectional communication between the Church and state authorities (43). By contrast, when dialogue was absent or insufficiently developed, the epidemic control measures were partly ineffective, and their potential to mitigate the spread of infection was not fully achieved.

Engaging faith-based communities for the improvement of public health is often challenging due to the complexities which arise from the implications of the implemented policies (11, 12, 46). The examples described in this paper reveal how active consultation between state authorities and religious institutions is paramount to achieving the desired goals. At the same time, we show that the failure to acknowledge potential religious consequences may lead to a diminished impact of the measures and to lower public compliance with official guidelines. The principles outlined herein extend to a variety of public health contexts, irrespective of country or religious belief. Therefore, we comprised several general policy recommendations which can be applied to improve the health of communities worldwide.

First, establishing clear guidelines is a necessary prerequisite for the success of any public health intervention. Public compliance and trust in healthcare authorities are both dependent on evidence-based policies that are implemented with transparency. As such interventions generally have consequences on multiple strata of society, an interdisciplinary approach is best suited to ensure that public health interventions have an enhanced impact. When religious communities are involved, this becomes particularly important due to any potential theological implications. Therefore, respectful consideration of any effects on religious practices both prior to and during the implementation phase may significantly increase the compliance of the public and maximize outcomes for public health.

Second, it is essential to develop efficient dialogue with religious institutions and faith-based communities. As a starting point, pre-existing channels of communication in various parts of society should be identified. These can lay the foundation for efficient streams of important and timely information. Concurrently, meaningful consultations with representatives of religious institutions during all stages of policy implementation can significantly bolster the effect of public health measures. Importantly, once established, these channels of communication with religious communities should not be restricted to the context of emergencies and can subsequently be harnessed for other public health initiatives.

Third, policy-makers should provide appropriate guidelines on how to safely resume religious practices. Transparency of the decision-making process and comprehensive information on the implemented measures are paramount. Importantly, the religious leaders and faith-based groups which they represent will require detailed explanations of the ongoing situation in non-specialist language. Such resources will then allow them to provide feedback about how the implemented measures impacted their communities. This will ensure higher compliance with the measures that directly or indirectly impact religious activities whilst also respecting the necessary health and safety requirements. Concurrently, if the feedback is considered in an appropriate manner, state authorities will be able to adapt the public health policies in order to maximize their effect.

Together, these approaches will enable religious institutions and faith-based communities to contribute toward shared goals during all stages of public health policy implementation. Moreover, effective communication will prevent avoidable disruptions to individual freedoms, thereby resulting in even higher levels of public compliance. Synergistic effects can thus be achieved which can substantially increase the benefit for society and public health.

## Conclusion

The lessons learned from the involvement of the ROC throughout the COVID-19 pandemic demonstrate the importance of efficient bidirectional communication with faith-based institutions for the betterment of public health. At the same time, they illustrate both the main challenges in engaging religious institutions and the best approaches for reaching shared goals. Importantly, this case study reveals general principles of the interactions between governmental bodies and faith-based communities regarding the implementation of public health policies. The examples provided clearly highlight the mutual benefit of dialogue when public health measures impact on religious practices. Furthermore, they serve as a basis for the outlined policy recommendations, which readily apply to a variety of public health contexts. Indeed, through respectful consultations and comprehensive discussions with representatives of faith-based communities, the outcomes of many public health initiatives could be greatly enhanced in the future.

## Data Availability Statement

The original contributions presented in the study are included in the article/supplementary material, further inquiries can be directed to the corresponding author/s.

## Author Contributions

SD was involved in public health efforts in Romania and acted as an external intermediary between state authorities and religious representatives on several occasions throughout the COVID-19 pandemic. SD and MB coordinated the academic initiative underlying this work. All authors contributed to the writing of the manuscript.

## Funding

SD and MG are funded by the BBSRC, grant number BB/M011224/1.

## Conflict of Interest

The authors declare that the research was conducted in the absence of any commercial or financial relationships that could be construed as a potential conflict of interest.

## Publisher's Note

All claims expressed in this article are solely those of the authors and do not necessarily represent those of their affiliated organizations, or those of the publisher, the editors and the reviewers. Any product that may be evaluated in this article, or claim that may be made by its manufacturer, is not guaranteed or endorsed by the publisher.
